# Three-Dimensional Registration for Handheld Profiling Systems Based on Multiple Shot Structured Light

**DOI:** 10.3390/s18041146

**Published:** 2018-04-09

**Authors:** Shirazi Muhammad Ayaz, Danish Khan, Min Young Kim

**Affiliations:** 1 School of Electronics Engineering, IT College, Kyungpook National University, 1370 Sankyuk-dong, Buk-gu, Daegu 41566, Korea; m_ayaz_shirazi@yahoo.com (S.M.A.); danish@ee.knu.ac.kr (D.K.); 2 Research Center for Neurosurgical Robotic System, Kyungpook National University, 1370 Sankyuk-dong, Buk-gu, Daegu 41566, Korea

**Keywords:** multi-view registration, multiple shot structured light, handheld 3D scanning, coarse registration, registration refinement, visual navigation

## Abstract

In this article, a multi-view registration approach for the 3D handheld profiling system based on the multiple shot structured light technique is proposed. The multi-view registration approach is categorized into coarse registration and point cloud refinement using the iterative closest point (ICP) algorithm. Coarse registration of multiple point clouds was performed using relative orientation and translation parameters estimated via homography-based visual navigation. The proposed system was evaluated using an artificial human skull and a paper box object. For the quantitative evaluation of the accuracy of a single 3D scan, a paper box was reconstructed, and the mean errors in its height and breadth were found to be 9.4 μm and 23 μm, respectively. A comprehensive quantitative evaluation and comparison of proposed algorithm was performed with other variants of ICP. The root mean square error for the ICP algorithm to register a pair of point clouds of the skull object was also found to be less than 1 mm.

## 1. Introduction

Three dimensional measurements are popular in computer vision owing to their applications in medical and scientific imaging, reverse engineering, security, cultural heritage, industrial inspection and 3D map building. Several techniques, e.g., laser ranging, structured light, and passive stereo vision can be utilized for 3D range data acquisition. As a result of the rapid development of these sensing techniques, scientists and researchers have taken great interest in the multiview 3D reconstruction of real objects. The general procedure of generating 3D models of an object includes the acquisition of 3D data from different viewpoints (partial 3D shapes of the object) and integration of these point clouds into a 3D model. The complete process to generate the 3D model from several partial views is known as multiview 3D reconstruction.

Approaches for 3D reconstruction [1,2,3] based on passive stereo vision have been proposed in the literature. These approaches pose the correspondence problem [4] when the scenes or images lack sufficient texture on the surface of the 3D object. This problem was resolved by using structured light techniques [5,6,7] in which a projector (or projection system) replaces one of the cameras in the stereo pair and a coded pattern is projected onto the 3D object. User may not be able to acquire the complete 3D model from the modeling system in a single measurement step owing to self-occlusion and a limited field of view and requires merging multiple views into a complete 3D model [8]. To merge different range images, we need to align these scans with respect to a common coordinate system using the process known as registration. Multi-view 3D registration is very popular due to its applications in different fields, e.g., human body detection, 3D object scanning, 3D localization and ego-motion estimation. 

Multiview 3D reconstruction may be classified into two categories [9]: the first uses a fixed sensor for an object performing handheld motion, and the other uses a handheld scanner for a fixed object. Let us consider the handheld rotation of an object by a small angle, wherein the images of the object are captured by a fixed sensor; here the range images can be aligned using a refinement algorithm owing to the fixed camera coordinate system, whereas for the same rotation of handheld scanner, this assumption is not valid owing to large displacement of the object in two different views and to the change in camera coordinate system [9]. Hence the multiview registration approach to tackle handheld operation, consists of two stages, coarse registration and refinement using the iterative closest point (ICP) algorithm [8,9]. The coarse registration is needed to handle unstable handheld motion and especially for tackling large motion where ICP-based refinement does not perform well. The coarse registration is used to estimate the initial parameters of the camera pose and then the refinement technique is applied on the pair of coarsely registered 3D datasets. In case of the failure of the refinement stage owing to unstable handheld motion, the multiview 3D reconstruction utilizes the fast coarse registration stage. If the coarse registration transforms the 3D data using an accurate pose, the refinement stage starts registering the 3D point clouds again following a coarse-to-fine strategy [9]. 

Researchers have intensively studied the problem of registration of 3D shapes in the last two decades. Readers may find the details of these studies in reviews [10,11]. Registration problems can be classified into two categories: pairwise registration (local method) or multiview registration (global method) [12]. Pairwise registration may be defined as the registration of the overlapping views and the user may formulate the problem as the sum of squared distances provided that the 3D correspondences are known. The locally aligned range images using pairwise registration may be integrated into the 3D model, leading to loop closure problem, which may be resolved using global method known as multiview registration. The comparision of the local and global methods [13] is given under the following features: (1) Local methods perform the registration on the pair of point clouds in an iterative manner while the global methods consider all the point clouds matching key geometric features among them and generate an optimum solution using RANSAC (Random sample consensus) frame work; (2) Local methods need good initial solution for their better performance and global methods donot require any good initialization, but they face the problem of incorrect and insufficient matched features; (3) As the global methods suffer from the problem of incorrect and insufficient correspondences, local methods can be used to refine the registration yielded by the global methods. 

In rigid registration, we can model the transformation between the point clouds using 6 degrees of freedom (DOF). Researchers employed the registration approaches based on either the Singular Value Decomposition (SVD) [14] or the Principal Component Analysis (PCA). The literature also reported the registration based on the advanced iterative scheme using the ICP algorithm [15]. Researchers have proposed several variants of ICP, which are non-linear ICP [16], generalized ICP [17], and non-rigid ICP [18]. The user may select any of these variants of the ICP algorithm depending on several characteristics, which are accuracy, convergence rate, robustness and computation time. All these characteristics depend on the application of interest, 3D data and the imaging environment.

In this paper, the registration approach for the 3D handheld profiling system based on stereo vision and multiple shot structured light is proposed. This system consists of a stereo camera and a non-calibrated projector [19] and finds application in the 3D modeling and the reconstruction of the 3D objects. The proposed approach can be divided into three steps i.e., the two view 3D reconstruction based on active stereo vision, estimation of the relative translation and rotation for different views using visual navigation and multi-view registration based on the ICP algorithm. The remainder of this paper is organized as follows: Section 2 describes the methodology of the proposed research. In Section 3, we discuss the experiments and results conducted using the proposed approach of the 3D registration and the 3D profiling system based on multiple shot structured light. Section 4 concludes this research and also provides the directions for future work.

## 2. Materials and Methods

The proposed method can be described into three stages: proposed approach, two view 3D reconstruction and multi-view 3D reconstruction.

### 2.1. Proposed Approach

The proposed handheld profiling system consists of a stereo camera and a non-calibrated illumination projector employed for 3D modelling, which is different from camera-projector based systems [4,19]. The 3D sensing systems [20,21,22,23,24] are related to the proposed 3D sensing system, but are employed for single view geometry. We have previously reported a procedure for the 3D reconstruction for variable zoom using stereo vision and structured light [25], but it was based only on the single view 3D reconstruction. The proposed hardware of the handheld system comprises of stereo camera and non-calibrated projector without zoom lenses, and the multiview 3D registration is proposed in this paper. This research is an extension of the previous work [25] on the 3D reconstruction; it enhances the accuracy of the 3D reconstruction using a multi-view procedure. The proposed approach belongs to the multiview 3D reconstruction consisting of the handheld system for a fixed 3D object. Owing to large motion and the change in camera coordinate system in our case, the multi-view registration based on coarse registration and pairwise ICP based final refinement is proposed. The final refinement based on the ICP algorithm depends on the coarse registration stage. If the coarse registration transforms the point clouds using accurate visual navigation parameters, the refinement stage further enhances the accuracy of the 3D model. The stereo camera system consisted of two cameras (acA2500-14gm, Basler, Exton, PA, USA). The projector used in this work was an mini beam PA75K (LG, Daejeon, Korea).

**Figure 1:**
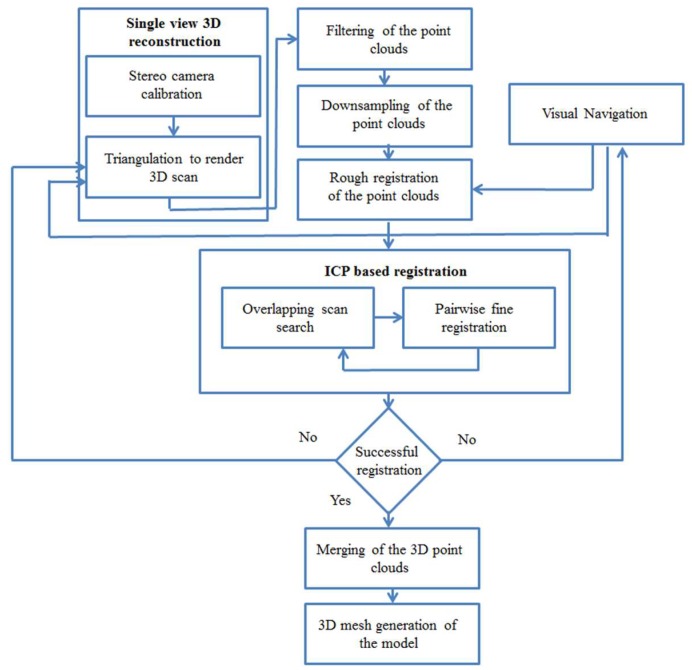
The block diagram of the proposed approach showing different algorithms.

Passive stereo vision-based 3D imaging poses the correspondence problem owing to less texture in a scene. To solve this problem, structured light technique is used to create artificial texture in the resulting images [20]. The block diagram of the proposed approach is shown in Figure 1 which depicts the flow of different algorithms in this research. 

### 2.2. Two View 3D Reconstruction

The two view 3D reconstruction approach is similar to the method described in [25], which utilizes the binary coded structured light and normalized cross correlation (NCC) for pattern projection and stereo matching respectively. The calibration object used in the current study was a 7 × 6 chessboard target. The algorithm in [26] was employed for corner detection of the chessboard target.

In the current research, the binary coded multiple shot structured light technique was used to acquire 3D scans for the handheld profiling system. A lot of research has been performed in the 3D handheld scanning field, but these approaches utilized the single shot structured light technique. To the best of our knowledge, there are no reports of handheld scanning approaches utilizing a multiple shot structured light technique. If the target 3D object is static and the application does not impose stringent constraints on the acquisition time, multiple-shot techniques can be used and may often result in more reliable and accurate results. However, if the target is moving, single-shot techniques have to be used to acquire a snapshot 3D surface image of the 3D object at particular time instance [27]. A high speed and low-cost approach for structured light pattern sequence projection using a fast rotating binary spatial light modulator is reported in [28]. This system has the capability to yield high accuracy measurements at 200 Hz of the projection frequency and 20 Hz of the 3D reconstruction rate. The research reported in [29] describes the system, which consists of a projector that is held in one hand and a fixed camera, that captures the 3D object’s geometry in less than 1 s using pattern sequence projection and reconstructs it in less than 30 s on a desktop computer. This approach may also be extended to obtain the representation of a whole object and align the different view point clouds using the ICP algorithm [29]. According to the research studies in [28,29], handheld scanning based on multiple shot structured light is feasible if a special hardware is utilized and the pattern projection and capturing processes take less time compared to handheld motion. Our system is also different from that reported in [29]. The proposed hardware is also capable of projecting and capturing binary patterns in less than 1 s for single scan.

The system’s calibration consists of two stages: pre-calibration based on Zhang’s method [30] and stereo camera calibration based on linear least square technique [31]. Pre-calibration was performed using images captured at a specific distance from system to remove distortion from the images at different distances. The undistorted image data can be obtained using Equation (1) from [32]:

(1) $$\left[\begin{array}{c} x_{p}\\ y_{p} \end{array}\right]=\left(1+k_{1}r^{2}+k_{2}r^{4}\right)\left[\begin{array}{c} x_{d}\\ y_{d} \end{array}\right]+\left[\begin{array}{c} 2p_{1}x_{d}y_{d}+p_{2}(r^{2}+2x_{d}^{2})\\ p_{1}(r^{2}+2y_{d}^{2})+2p_{2}x_{d}y_{d} \end{array}\right]$$

where (*x_p_*, *y_p_*) and (*x_d_*, *y_d_*) are the corrected and distorted pixel coordinates, respectively.

For two view 3D reconstruction, the fundamental matrix was estimated from Equation (2) using random sample consensus (RANSAC) algorithm [33]. Binary coded structured light was used to obtain the coded images of the stereo camera and NCC was utilized for stereo matching [25] in this research. Binary patterns were projected on the 3D projected and captured by the proposed hardware. The procedure for generation of binary coded images from the projected binary patterns for the handheld profiling system is as follows: (1) Nine images for each camera are loaded, which consist of one all-white, one all-black, and seven binary images; (2) The average of all-white and all-black images is determined and compared with the binary images; (3) Each pixel in the binary images is examined to determine whether it is illuminated or not by thresholding; (4) The code from all binary images is concatenated into a binary coded image:

(2) $$q_{r}^{T}Fq_{l}=0$$

Epipolar geometry can be defined as the basic geometry of the stereo camera which describes the relationship between the image coordinates of the stereo pair. Some facts about the epipolar geometry [32] are listed as follows: (1) Epipolar plane contains every 3D point visible in both cameras and it intersects each image in stereo pair in an epipolar line; (2) For feature point given in the left image, the matched feature must be located along the corresponding epipolar line and this constraint is termed as epipolar constraint; (3) Epipolar constraint converts the two dimensional search for stereo matching into one dimensional along the epipolar lines provided that the epipolar geometry of the stereo camera is known; (4) Therefore, epipolar constraint reduces the computation expenses of the stereo matching and excludes the features that may result in false matches; (5) For the two feature points visible in the field of view of both cameras appearing in a specific order in the left image, the correspondences of these points in the right image will occur in the same order as of the left image. 

**Figure 2:**
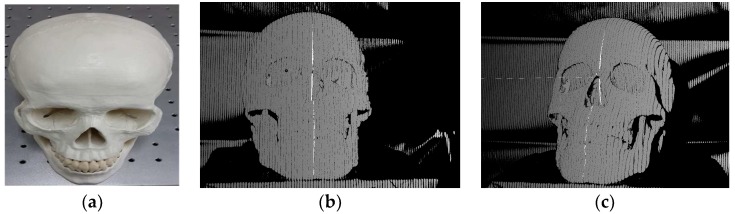
Description of the epipolar geometry in the binary coded images using the skull object, (**a**) the actual skull object used for the 3D reconstruction; (**b**) the left image shows the point indicated as black circle; and (**c**) the right image shows epipolar line passing through the same position as of dark circle indicated in the left image.

The binary coded images were used in the stereo matching based on NCC and the whole images are processed to render the 3D point cloud for a single scan. Since this matching process consumes huge time, the region of interest (ROI) of the stereo pairs enclosing the 3D object was used for yielding the 3D data, which improves the computation time. We demonstrated the epipolar geometry between the binary coded images in the stereo pair shown in Figure 2. Figure 2b,c depict binary coded images of the left camera with the point indicated by black circle and the coded image of the right camera shows the epipolar line passing through the matched point between the stereo pair; whereas the actual skull object used for the 3D reconstruction is depicted in Figure 2a.

### 2.3. Multiview 3D Reconstruction

Multi-view 3D reconstruction of the point cloud data consists of two stages: rough registration and fine registration based on the ICP algorithm. Rough registration is based on camera parameters estimated using visual navigation. For large and unstable handheld motion, a coarse-to-fine strategy is employed in multiview 3D reconstruction.

The visual navigation algorithm determines the relative rotation and translation parameters of a single moving camera using RANSAC-based homography estimation [32,33]. The projective mapping between the two images or planes is known as homography. The relationship between the image points in the source and destination images is expressed by a homography matrix ‘*H*’. The Direct Linear Transform (DLT) algorithm can be used to estimate the matrix ‘*H*’ using sufficient number of matched features [34] as given below:

(3) $$c\left(\begin{array}{c} u\\ v\\ 1 \end{array}\right)=H\left(\begin{array}{c} x\\ y\\ z \end{array}\right)$$

where:

$$H=\left(\begin{array}{ccc} h_{11} & h_{12} & h_{13}\\ h_{21} & h_{22} & h_{23}\\ h_{31} & h_{32} & h_{33} \end{array}\right)$$

After dividing the first row and the second row of Equation (3) by the third row, we get the following two equations:

(4) $$-h_{11}x\;-\;h_{12}y\;-\;h_{13}\;+\;\left(h_{31}x\;+\;h_{32}y\;+\;h_{33}\right)u\;=\;0$$

(5) $$-h_{21}x\;-\;h_{22}y\;-\;h_{23}\;+\;\left(h_{31}x\;+\;h_{32}y\;+\;h_{33}\right)v\;=\;0$$

Equations (4) and (5) can be written in matrix form as follows:

(6) $$A_{i}h\;=\;0$$

$$A_{i}=\left(\begin{array}{cccc} \begin{array}{c} -x\\ 0 \end{array} & \begin{array}{c} -y\\ 0 \end{array} & \begin{array}{c} -1\\ 0 \end{array} & \begin{array}{cccc} \begin{array}{c} 0\\ -x \end{array} & \begin{array}{c} 0\\ -y \end{array} & \begin{array}{c} 0\\ -1 \end{array} & \begin{array}{cc} \begin{array}{c} ux\\ vx \end{array} & \begin{array}{cc} \begin{array}{c} uy\\ vy \end{array} & \begin{array}{c} u\\ v \end{array} \end{array} \end{array} \end{array} \end{array}\right)$$

$$h={\left(\begin{array}{cccc} h_{11} & h_{12} & h_{13} & \begin{array}{cccc} h_{21} & h_{22} & h_{23} & \begin{array}{ccc} h_{31} & h_{32} & h_{33} \end{array} \end{array} \end{array}\right)}^{T}$$

The pose and position of a single moving camera may be determined via homography decomposition provided that the intrinsic camera matrix is known. The equations for the homography estimation and decomposition are as follows:

(7) $$b_{dst}=Hb_{src}$$

(8) $$b_{src}=H^{-1}b_{dst}$$

$$b_{dst}=\left[\begin{array}{c} u_{dst}\\ v_{dst}\\ 1 \end{array}\right],\;b_{src}=\left[\begin{array}{c} u_{src}\\ v_{src}\\ 1 \end{array}\right]$$

(9) $$r_{1}=\lambda M^{-1}h_{1}$$

(10) $$r_{2}=\lambda M^{-1}h_{2}$$

(11) $$r_{3}=r_{1}\times r_{2}$$

(12) $$t=\lambda M^{-1}h_{3}$$

where (*u_src_*, *v_src_*) are the pixel coordinates of the source image; (*u_dst_*, *v_dst_*) are the pixel coordinates of the destination image; *H* = [*h*_1_ *h*_2_ *h*_3_] is a 3 × 3 homography matrix; *r_i_* is the *i*-th column of the 3 × 3 rotation matrix; *h_i_* is a 3 × 1 vector, *i* = 1 to 3; λ is the scaling factor; and M is the intrinsic matrix of the camera (known by calibration).

The rough registration is based on the transformation of the point clouds into the coordinate of the reference view using the parameters from visual navigation. The mathematical equation is given below:

(13) $$X_{i}^{ref}=R_{i}X_{i}+T_{i}$$

where *X_i_* is the 3D point cloud of the *i*-th view, *R_i_* is the relative rotation between the *i*-th view and the reference view point cloud, *T_i_* is the relative translation between the *i*-th view and the reference view point cloud, and *X_i_^ref^* is the *i*-th point cloud transformed into the reference view coordinate.

**Figure 3:**
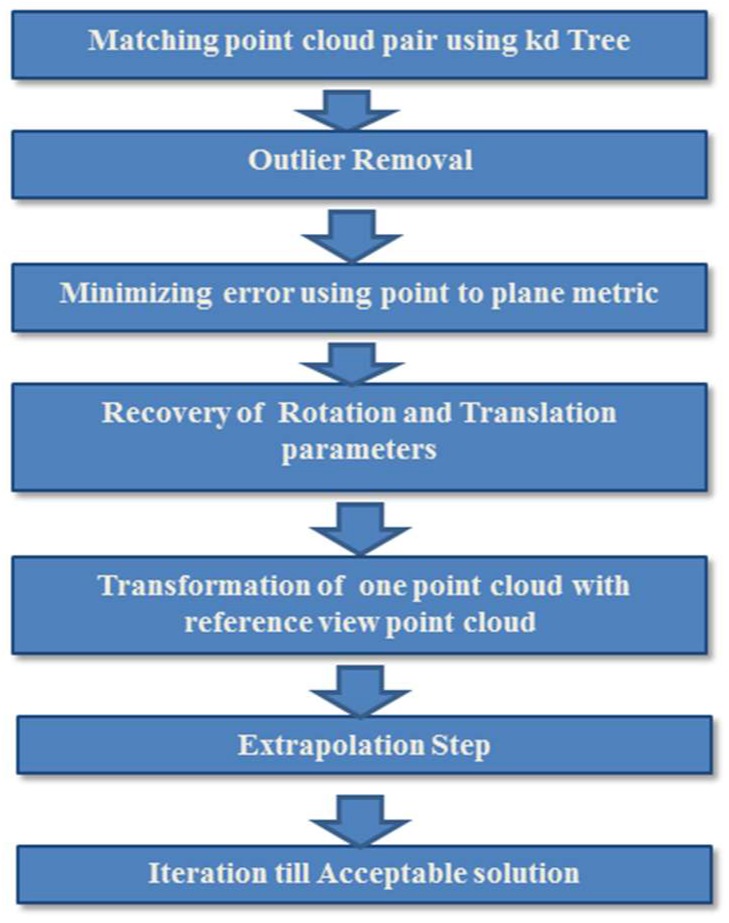
Block diagram of the ICP algorithm depicting different steps for point cloud refinement.

The points clouds were further refined using the ICP algorithm, which is a modified version of that presented in [16]. The ICP algorithm consists of an extrapolation step that traces out a path in the registration state space from the identity transformation toward a locally optimal shape match [15]. The extrapolation step results in reducing the number of iterations for fast convergence of the ICP algorithm. The mathematics of the proposed algorithm is similar to the algorithm presented in [16], but a number of modifications have been made. The ICP algorithm presented in [16] is based on minimizing the distance measure function derived from the definition of the 3D surface registration. This registration algorithm based on Levenberg Marquardt (LM) algorithm to solve least-square equations, is computationally expensive. To solve this problem, we did not employ the LM algorithm and an extrapolation step [15] has been added to further accelerate the proposed ICP algorithm. The proposed algorithm also consists of worst 10% of pairs-based outlier rejection method [35]. The algorithm in [15] needs a 3D model and a sensed model (data model) for the 3D registration, whereas the proposed algorithm does not need the 3D model of an object. The block diagram of ICP algorithm is depicted in Figure 3 using the reference view point cloud and the other viewpoint cloud. 

**Figure 4:**
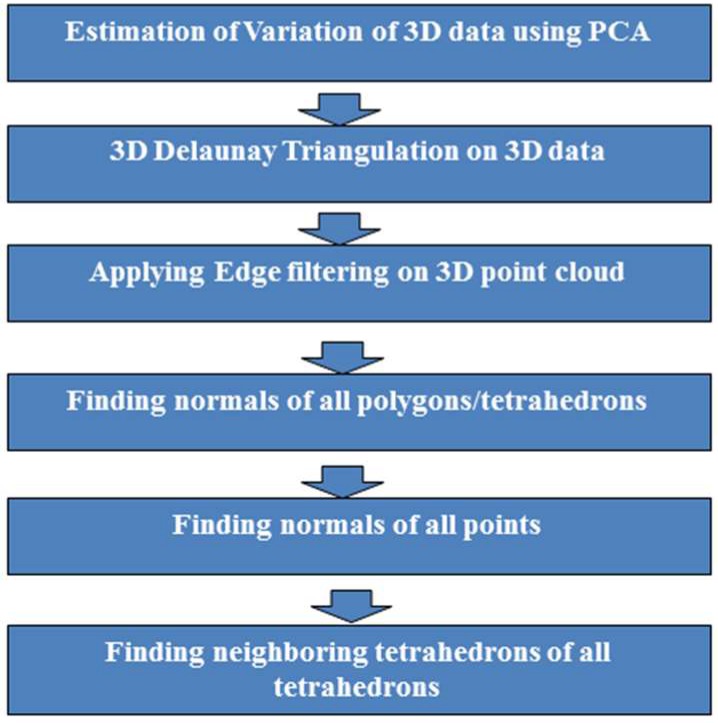
Block diagram of the algorithm for the formation of 3D mesh from point clouds.

The final refinement stage based on the ICP algorithm yields good accuracy if the initial estimation of pose for coarse registration is also accurate. If the initial pose has good accuracy, the refinement stage starts registering the 3D point clouds again following a coarse-to-fine strategy and yielding high registration accuracy. The block diagram of the algorithm for the formation of 3D mesh is shown in Figure 4.

## 3. Experiments and Results

This section describes the experiments and results of this research. Two objects were selected for the 3D reconstruction and were placed at 50 cm from the handheld profiling system. These objects included a skull, which was reconstructed as the qualitative demonstration of the 3D modeling, and a box, which was used to quantitatively analyze the 3D reconstruction results. Our previous research [25] has described the details of the experimental setup and has also mentioned the working distances and illumination patterns. That description is also applicable to this handheld scanning research. The studies in [25] utilized a zoom lens while the current research did not employ any zoom lens. The current research is based on multiview geometry while the study in [25] was based on single view geometry.

**Figure 5:**
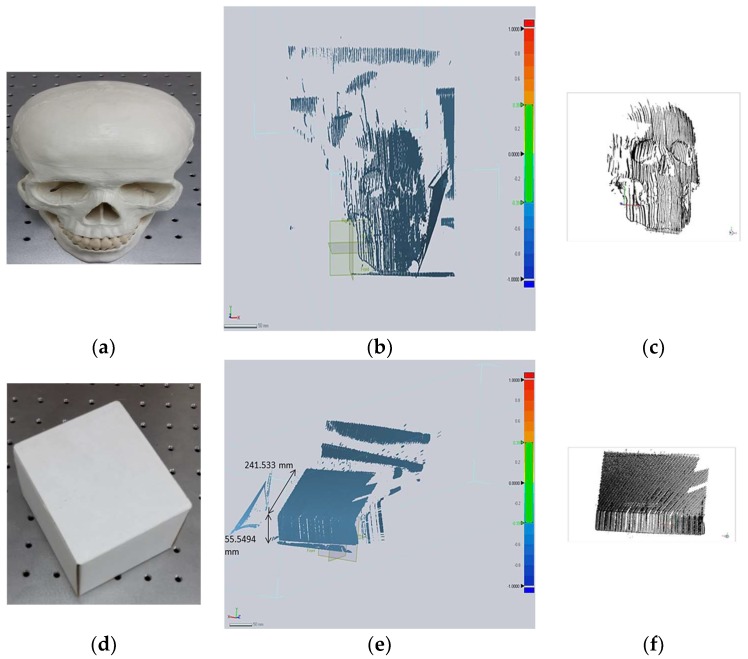
Results of the 3D reconstruction, (**a**–**c**) Qualitative analysis of the 3D reconstruction showing the actual skull object, and the preservation of the features of the skull before and after post processing, (**d**–**f**) Quantitative analysis of the 3D reconstruction showing the actual box object, its measured height and length and the result of the 3D reconstruction before and after the post processing.

First, the 3D reconstruction of the skull object, shown in Figure 5a, was carried out and the raw 3D point cloud was further processed using the Geomagic Control software (3D Systems, Inc., Rock Hill, SC, USA). The results of the 3D reconstruction of the skull before and after the post-processing of the point cloud are shown in Figure 5b,c. A single view 3D scan of the skull shows good quality of the point cloud with the preservation of features, the shape of the 3D object is also visible as depicted in Figure 5c and also in Figure 9a below, which shows the result of the mesh of another single view 3D point cloud of the skull.

We also performed the experiment using the box object, shown in Figure 5d, to evaluate the accuracy of a single view 3D reconstruction by measuring the height and length of the same object as the accuracy in a single view 3D reconstruction directly corresponds to the accuracy of the 3D modeling. Therefore, the paper box was placed at 50 cm from handheld profiling system. Figure 5e,f show the 3D reconstruction result of the box before and after post-processing of the point cloud. Table 1 shows the accuracy of the dimensions of the paper box object i.e., the mean errors in height and length of the box were found to be 9.4 μm and 23 μm, respectively, which demonstrate the good accuracy of the single view 3D reconstruction. Mean measured value is the mean of a specific number of manual measurements of the dimension of the paper box in Geomagic Verify viewer. Mean error is the difference between the original value and the mean measured value. This procedure is to show the quantitative evaluation of the accuracy of the single view 3D reconstruction and it is not related to ICP.

**Figure 6:**
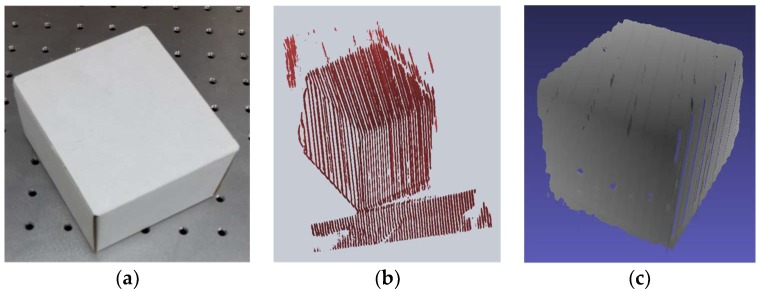
Results of the 3D reconstruction of a single view of the box object, (**a**) the actual box object; (**b**) Visualization of the 3D point cloud; and (**c**) Visualization of the mesh refined in the MeshLab software (ISTI, CNR, Pisa, Italy).

Figure 6 also shows the quality of the 3D reconstruction of the box object in Figure 6a. The result of the visualization of the point cloud of the box object for another single view is shown in Figure 6b, whereas Figure 6c depicts the refined mesh of the same view of the box object.

**Figure 7:**
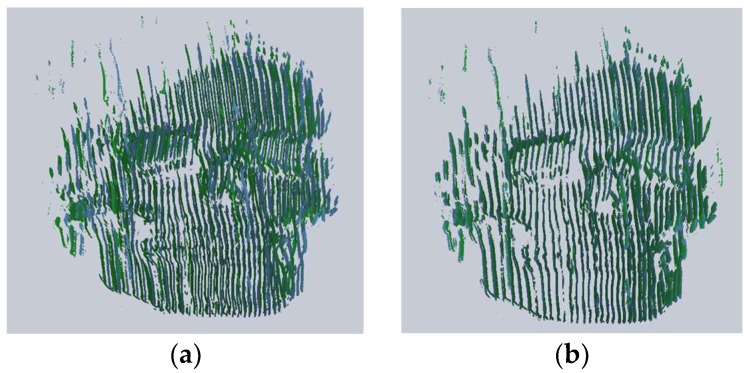

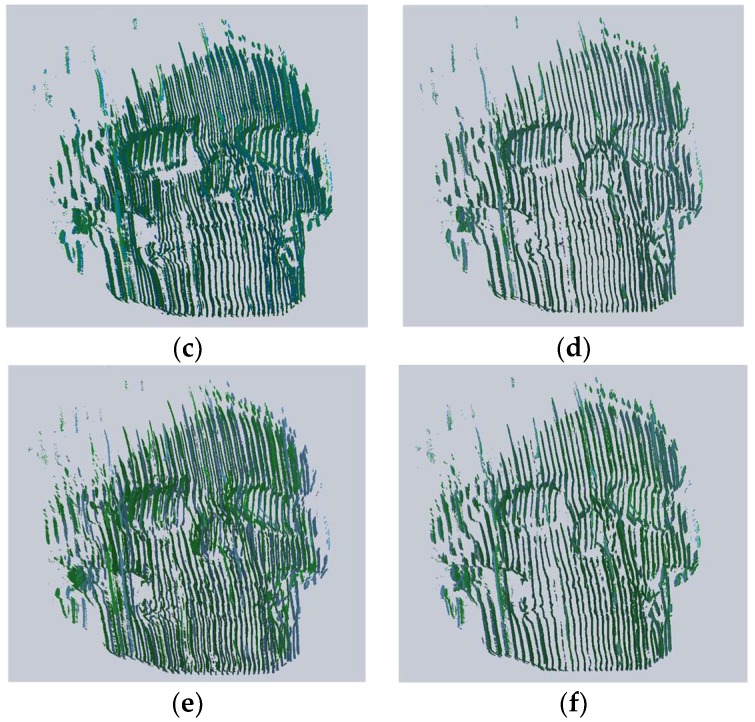
Visualization before and after applying the ICP algorithm (**a**,**b**) for first pair, (**c**,**d**) for 2nd pair and (**e**,**f**) for 3rd pair.

For the registration of the point clouds, the two view 3D reconstructions were performed using the skull for different views performing a handheld motion. A visualization of the two-point clouds of skull before and after applying the ICP algorithm shown in green and blue colors is depicted in Figure 7 for three pairs of roughly registered point clouds, which demonstrates final refinement using the ICP algorithm and further enhancement of the shape of the skull. Each pair consists of a reference view point cloud and a roughly registered view with respect to reference view via coarse registration stage. The root mean square error (RMS) for the three pairs of point cloud registered using the ICP algorithm is shown in Table 2; the table also shows the average of number of the 3D points of the two point clouds in each pair. The accuracy for the ICP algorithm-based final refinement in RMS is found to be less than 1 mm. 

**Figure 8:**
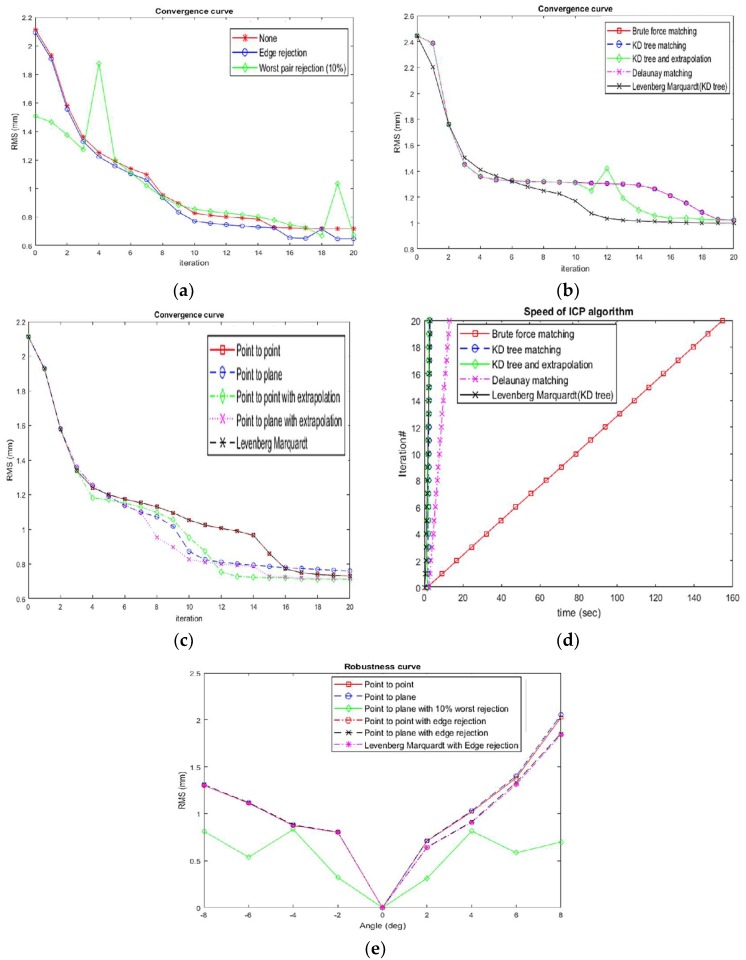
Characteristics of ICP to compare the proposed algorithm with the other variants, (**a**–**c**) the convergence behavior of the proposed algorithm and other variants for outlier removal, matching and error metrics respectively, and (**d**,**e**) speed and robustness of the proposed algorithm and other variants.

In order to evaluate the ICP algorithm quantitatively and compare it with other variants of ICP, we followed some of the procedures related to [35,36]. We compared the proposed ICP algorithm with other variants using the 3D point clouds produced by the 3D handheld scanning system based on multiple shot structured light and analyzed the accuracy, convergence behavior, speed and the robustness of the algorithms. Figure 8a shows the convergence behavior of the ICP variants for outlier rejection schemes i.e., worst 10% of pairs (proposed one), edge rejection and no outlier rejection. The graph shows that the edge rejection outperforms the other schemes while worst pair rejection performs close to the edge rejection scheme. Five matching strategies, i.e., brute force matching, K-D tree matching, K-D tree and extrapolation (proposed one), Delaunay matching and Levenberg Marquardt (LM) with K-D tree, were compared using the 3D data produced by the proposed system in Figure 8b for convergence behavior. The results show that LM algorithm with K-D tree performed better than the other strategies, while the convergence behavior of K-D tree with extrapolation was close to the LM (K-D tree) and other variants. The overshoot observed in case of the K-D tree with extrapolation is mainly due to extrapolation step [35]. Five error metrics, point to point, point to plane, point to point with extrapolation, point to plane with extrapolation (proposed one) and point to point using LM algorithm, were comparatively analyzed in Figure 8c. The results demonstrate that the convergence behavior of the extrapolated point to point and point to plane error metrics are the same or better than those of the other metrics, while the point to point and the point to point with LM algorithm performed better than the point to plane metrics. The convergence behaviour of the point to point with LM and the point to point metric is same as the point clouds have good overlap. 

In order to evaluate the speed of the proposed algorithm with other ICP variants, matching strategies, brute force matching, K-D tree matching, K-D tree and extrapolation (proposed one), Delaunay matching and Levenberg Marquardt (LM) with K-D tree, were compared using the two 3D point clouds of each of 40–42 k data points shown in Figure 8d. The graph concludes that the brute force matching is the most computationally expensive matching scheme and the performance of the K-D tree based matching schemes is similar. Among the K-D tree-based schemes, K-D tree with extrapolation outperforms the other matching strategies. Since the point clouds have good overlap, K-D tree with LM algorithm performs similar to the K-D tree matching. In order to evaluate the accuracy of the proposed algorithm and ICP variants, we fixed the Handheld scanner on a rotational stage and acquired the 3D point clouds at five angles with 2 degrees difference between the consecutive point clouds. After applying the rough registration, we applied the different strategies for ICP, i.e., point to point, point to plane, point to plane with 10% worst rejection (proposed one), point to point with edge rejection, point to plane with edge rejection and point to point with LM algorithm (with edge rejection). The results were recorded in Table 3 and the best angle measurements are shown in bold. In case of the point to point based strategies, the point to point based angle measurements were improved using edge rejection and LM algorithm. While the point to plane with edge rejection performs better than the point to plane and point to plane with 10% worst pair rejection. Finally, the point to plane with edge rejection based angle measurements are more accurate than those with point to point with edge rejection. To evaluate the robustness of the proposed algorithm, we performed the acquisition of the point clouds at 8 to −8 degree with the step size of 2 and the point cloud at 0 degree is taken as the reference point cloud for all the other point clouds [36,37]. We compared error metrics with rejection strategy, i.e., point to point, point to plane, point to plane with 10% worst rejection (proposed one), point to point with edge rejection, point to plane with edge rejection, and point to point with LM and edge rejection as shown in Figure 8e. The results show that the point to plane with 10% worst rejection outperforms the other error metrics and follows a symmetry on either side of ‘zero’ degree position. The point to point, the point to point with LM and point to plane error metrics with edge rejection performed better than the point to point and point to plane error metrics without edge rejection on the point clouds at positive angles.

**Figure 9:**
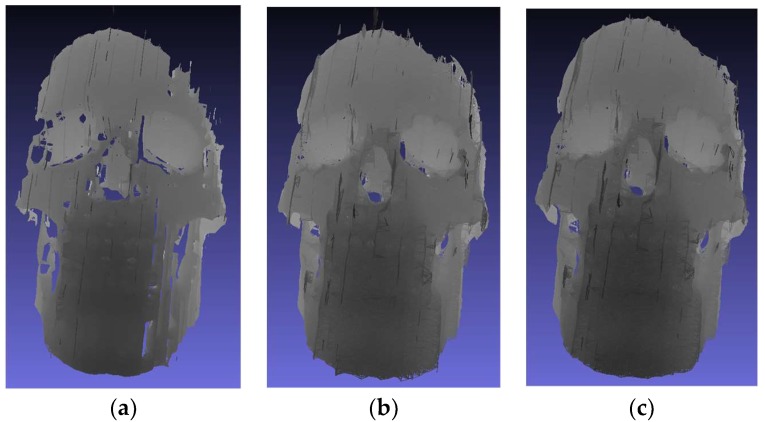
Results of the 3D mesh formation (**a**) Raw mesh for a single view (**b**) Raw mesh for the integration of five point clouds registered after the ICP algorithm (**c**) Refined mesh using the MeshLab software for the integration of five point clouds registered after the ICP algorithm.

The 3D mesh for one view and the integration of five views after applying the ICP algorithm are shown in Figure 9a–c. Figure 9a shows the visualization of a single view mesh depicting the good quality of the single view 3D reconstruction, whereas the mesh of the integration of five views before and after refinement using the MeshLab software (University of Pisa, Italy) [38], is depicted in Figure 9b,c. The results of the 3D mesh generation shown in Figure 9a,b, demonstrate the difference between the mesh of the single view point cloud and mesh of the integration of five point clouds in term of holes. The holes in Figure 9a have been compensated as shown in Figure 9b using the proposed multiview 3D reconstruction. In order to find the surface divergence between the merged point clouds (five point clouds) and the 3D model of the skull phantom, we generated the 3D replica of the skull phantom using a 3D scanner (DAVID SLS-3; Hewlett-Packard, Palo Alto, CA, USA) having an accuracy of 50 µm and we performed the comparison between the merged point clouds with the 3D model using CloudCompare software [39]. The 3D Scanner used as a reference platform is the structured light 3D scanner (DAVID SLS-3). The specifications of this scanner are as follows:

- Scan size: 60–500 mm
- Resolution/Precision: Up to 0.05% of scan size (up to 0.05 mm)
- Scanning time: One single scan within a few seconds
- Mesh density: Up to 2,300,000 vertices per scan
- Export formats: OBJ, STL, PLY

**Figure 10:**
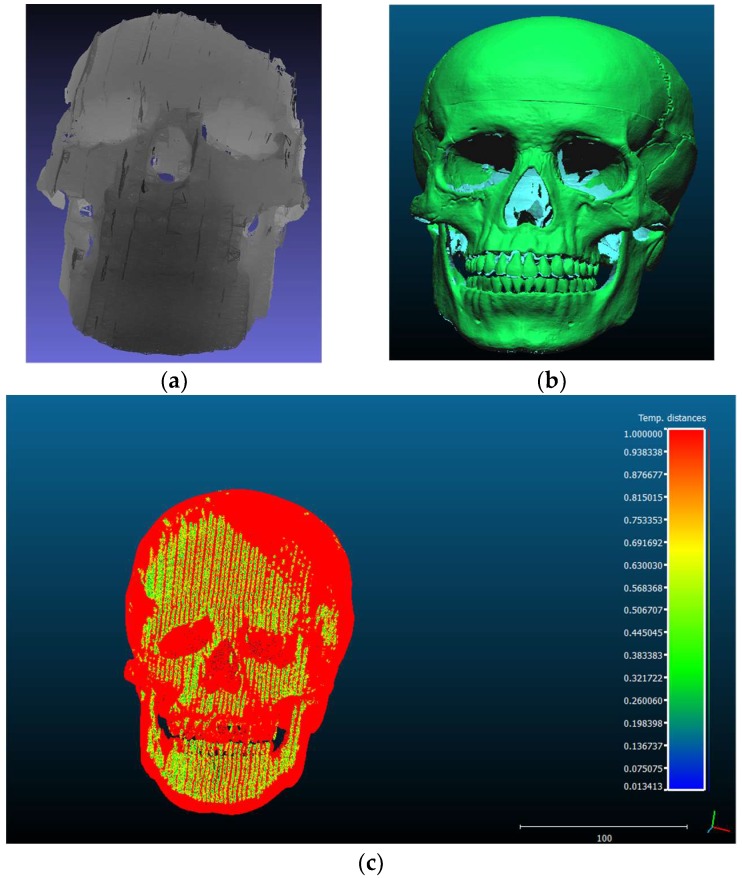
Surface divergence result between the merged point clouds and the 3D model (**a**) merged point clouds of the proposed system; (**b**) the 3D model generated using the 3D scanner; (**c**) Surface divergence estimation between the merged point clouds and the 3D model using CloudCompare software.

Figure 10a–c show the merged point clouds, 3D model and the surface divergence between the merged point clouds and the 3D model respectively. The mean distance between the merged point clouds and the 3D model was 0.94 mm while the standard deviation was found to be 0.15 mm. 

## 4. Conclusions

In this paper, we have implemented a 3D handheld profiling system based on multiview stereo vision and multiple shot structured light. The system consists of the handheld profiling system using a stereo camera and a non-calibrated projector. Single view 3D reconstruction approach based on binary coded structured light and NCC was utilized to get the point clouds of different views. 

A rough registration of multiple point clouds was performed using the relative orientation and translation parameters estimated via homography-based visual navigation. The registered point clouds were further refined using the ICP algorithm. The system was tested using an artificial human skull and a paper box object to demonstrate the qualitative and quantitative analysis of the 3D reconstruction. For the quantitative evaluation of the proposed system, a paper box was reconstructed and errors in its height and breadth were found to be 9.4 μm and 23 μm, respectively. A comprehensive quantitative evaluation and comparision of the proposed algorithm was performed with other variants of ICP. The proposed ICP algorithm was found to be comparable to the other variants of ICP. The mean distance between the merged point clouds and the 3D model was 0.94 mm while the standard deviation was found to be 0.15 mm. Future research directions include the modelling of human body parts and the utilization of a single shot binary pattern to reduce the processing time. The processing time can be further reduced using parallel processing. 

## Figures and Tables

**Table 1:** Results of the 3D reconstruction of the paper box to demonstrate the accuracy of single view reconstruction.

S.No.	Object Dimension	Original Value (mm)	Mean Measured Value (mm)	Mean Error (μm)
1.	Height	55.54	55.5494	9.4
2.	Length	241.51	241.533	23

**Table 2:** RMS for the ICP algorithm applied for three pairs of roughly registered point clouds.

S.No.	Pair of Point Clouds	Average 3D Points of Point Clouds	RMS for ICP Algorithm (mm)
1.	First	883,116	0.7143
2.	Second	881,165	0.4990
3.	Third	875,165	0.7621

**Table 3:** Accuracy of the proposed ICP algorithm and its comparison with the other variants.

S.No.	ICP Variants	Measured Angle (deg)				Ground Truth Angle (deg)			
1.	Point to point	2.29	3.64	6.70	8.90	2	4	6	8
2.	Point to plane	2.44	3.74	6.51	8.49	2	4	6	8
3.	Point to plane (10% worst rejection)	2.43	3.66	6.35	8.94	2	4	6	8
4.	Point to point with edge rejection	2.26	3.58	6.66	8.78	2	4	6	8
5.	Point to plane with edge rejection	2.36	3.67	6.49	8.45	2	4	6	8
6.	LM with Edge rejection	2.26	3.58	6.66	8.78	2	4	6	8

## References

[1] Ram V., Zhou Z., Kurillo G., Lobaton E., Bajcsy R., Nahrstedt K. Real-time stereo-vision system for 3D teleimmersive collaboration. Proceedings of the 2010 IEEE International Conference on Multimedia and Expo (ICME).

[2] Stavros H., Ttofis C., Georghiades A.S., Theocharides T. Towards hardware stereoscopic 3D reconstruction: A real-time FPGA computation of the disparity map. Proceedings of the Conference on Design, Automation and Test in Europe, European Design and Automation Association.

[3] Aissaoui A., Martinet J., Djeraba C. (2014). Rapid and accurate face depth estimation in passive stereo systems. Multimedia tools and applications. Multimed. Tools Appl..

[4] Salvi J., Pages J., Batlle J. (2004). Pattern codification strategies in structured light systems. Pattern Recognit..

[5] Ben-Hamadou A., Soussen C., Daul C., Blondel W., Wolf D. (2013). Flexible calibration of structured-light systems projecting point patterns. Comput. Vis. Image Underst..

[6] Rayas J.A., León-Rodríguez M., Martínez A., Genovese K., Medina O.M., Cordero R.R. (2017). Using a single-cube beam-splitter as a fringe pattern generator within a structured-light projection system for surface metrology. Opt. Eng..

[7] Wijenayake U., Park S.Y. (2015). Dual pseudorandom array technique for error correction and hole filling of color structured-light three-dimensional scanning. Opt. Eng..

[8] Ayaz S.M., Kim M.Y. (2017). Multiview registration-based handheld 3D profiling system using visual navigation and structured light. Int. J. Optomech..

[9] Park S.-Y., Baek J., Moon J. (2011). Hand-held 3D scanning based on coarse and fine registration of multiple range images. Mach. Vis. Appl..

[10] Tam G.K.L., Cheng Z.Q., Lai Y.-K., Langbein F.C., Liu Y., Marshall D., Martin R.R., Sun X.-F., Rosin R.L. (2013). Registration of 3D point clouds and meshes: A survey from rigid to nonrigid. VCG.

[11] Díez Y., Roure F., Lladó X., Salvi J. (2015). A qualitative review on 3D coarse registration methods. ACM Comput. Surv..

[12] Yizhi T., Feng J. (2015). Hierarchical multiview rigid registration. Comput. Graph. Forum.

[13] Geng J. (2011). Structured-light 3D surface imaging: A tutorial. Adv. Opt. Photon..

[14] Cloud Compare—3D Point Cloud and Mesh Processing Software—Open Source Project. http://www.danielgm.net/cc/.

[15] Salvi J., Fernandez S., Pribanic T., Llado X. (2010). A state of the art in structured light patterns for surface profilometry. Pattern Recognit..

[16] Fantoni S., Castellani U., Fusiello A. Accurate and Automatic Alignment of Range Surfaces. Proceedings of the 2012 Second International Conference on 3D Imaging, Modeling, Processing, Visualization and Transmission (3DIMPVT).

[17] Schaffer M., Grosse M., Kowarschik R. (2010). High-speed pattern projection for three-dimensional shape measurement using laser speckles. Appl. Opt..

[18] Bruno F., Bianco G., Muzzupappa M., Barone S., Razionale A.V. (2011). Experimentation of structured light and stereo vision for underwater 3D reconstruction. ISPRS J. Photogramm. Remote. Sens..

[19] Da A., Woodward A., Delmas P. Comparison of active structure lighting mono and stereo camera systems: Application to 3d face acquisition. Proceedings of the Seventh Mexican International Conference IEEE on Computer Science, 2006. ENC’06.

[20] Zhang Z. (2000). A flexible new technique for camera calibration. IEEE Trans. Pattern Anal. Mach. Intell..

[21] Sun W., Yang X., Xiao S., Hu W. Robust Recognition of Checkerboard Pattern for Deformable Surface Matching in Multiple Views. Proceedings of the High Performance Computing & Simulation (HPCS 2008) Conference.

[22] Bradski G., Kaehler A. (2008). Learning OpenCV: Computer Vision with the OpenCV Library.

[23] Cho H. (2005). Optomechatronics: Fusion of Optical and Mechatronic Engineering.

[24] Fischler M.A., Bolles R.C. (1981). Random sample consensus: A paradigm for model fitting with applications to image analysis and automated cartography. Commun. ACM.

[25] MeshLab. http://www.meshlab.net/.

[26] Hu E., He Y. (2009). Surface profile measurement of moving objects by using an improved π phase-shifting Fourier transform profilometry. Opt. Lasers Eng..

[27] Chen C.S., Hung Y.P., Chiang C.C., Wu J.L. (1997). Range data acquisition using color structured lighting and stereo vision. Image Vis. Comput..

[28] Kim Y.M., Ayaz M.S., Park J., Roh Y. (2014). Adaptive 3D sensing system based on variable magnification using stereo vision and structured light. Opt. Lasers Eng..

[29] Chen Y., Medioni G. (1992). Object Modelling by Registration of Multiple Range Images. Image Vis. Comput. Butterworth-Heinemann.

[30] Besl P.J., McKaym N.D. (1992). A Method for Registration of 3-D Shapes. IEEE Trans. Pattern Anal. Mach. Intell..

[31] Wissmann P., Forster F., Schmitt R. (2011). Fast and low-cost structured light pattern sequence projection. Opt. Express.

[32] Koch S. (2012). Development of a Mobile Projector Camera System for Structured Light Scanning. Ph.D. Thesis.

[33] Taguchi Y., Jian Y., Ramalingam S., Feng C. Point-plane SLAM for hand-held 3D sensors. Proceedings of the 2013 IEEE International Conference on Robotics and Automation (ICRA).

[34] Marden S., Guivant J. Improving the Performance of ICP for Real-Time Applications using an Approximate Nearest Neighbour Search. Proceedings of the Australasian Conference on Robotics and Automation.

[35] Segal A., Haehnel D., Thrun S. Generalized-ICP. Proceedings of the Conference: Robotics: Science and Systems.

[36] Amberg B., Romdhani S., Vetter T. Optimal step nonrigid icp algorithms for surface registration. Proceedings of the 2007 IEEE Conference on Computer Vision and Pattern Recognition CVPR’07.

[37] Kjer H.M., Wilm J. (2010). Evaluation of Surface Registration Algorithms for PET Motion Correction. Bachelor’s Thesis.

[38] Hartley R., Zisserman A. (2003). Multiple View Geomerty in Computer Vision.

[39] Rusinkiewicz S., Levoy M. Efficient variants of the ICP algorithm. Proceedings of the IEEE Third International Conference on 3-D Digital Imaging and Modeling.

